# The Anti-Inflammatory Effect and Structure of EPCP1-2 from *Crypthecodinium cohnii* via Modulation of TLR4-NF-κB Pathways in LPS-Induced RAW 264.7 Cells

**DOI:** 10.3390/md15120376

**Published:** 2017-12-01

**Authors:** Xiaolei Ma, Baolong Xie, Jin Du, Aijun Zhang, Jianan Hao, Shuxun Wang, Jing Wang, Junrui Cao

**Affiliations:** The Institute of Seawater Desalination and Multipurpose Utilization, SOA, Tianjin 300192, China; xiebaolong@tju.edu.cn (B.X.); dujin111@126.com (J.D.); meimei842858@163.com (A.Z.); phoenix328@163.com (J.H.); w_shuxun@126.com (S.W.); wangjing@126.com (J.W.)

**Keywords:** *Crypthecodinium cohnii*, exo-polysaccharide, anti-inflammatory, TLR4-MAPKs/NF-κB pathways

## Abstract

Exopolysaccharide from *Crypthecodinium cohnii* (EPCP1-2) is a marine exopolysaccharide that evidences a variety of biological activities. We isolated a neutral polysaccharide from the fermentation liquid of *Crypthecodinium cohnii* (CP). In this study, a polysaccharide that is derived from *Crypthecodinium cohnii* were analyzed and its anti-inflammatory effect was evaluated on protein expression of toll-like receptor 4 and nuclear factor κB pathways in macrophages. The structural characteristics of EPCP1-2 were characterized by GC (gas chromatography) and GC-MS (gas Chromatography-Mass Spectrometer) analyses. The molecular weight was about 82.5 kDa. The main chain of EPCP1-2 consisted of (1→6)-linked mannopyranosyl, (1→6)-linked glucopyranosyl, branched-chain consisted of (1→3,6)-linked galactopyranosyl and terminal consisted of t-l-Rhapyranosyl. The in vitro anti-inflammatory activity was representated through assay of proliferation rate, pro-inflammatory factor (NO) and expressions of proteins on RAW 264.7, the macrophage cell line. The results revealed that EPCP1-2 exhibited significant anti-inflammatory activity by regulating the expression of toll-like receptor 4, mitogen-activated protein kinases, and Nuclear Factor-κB protein.

## 1. Introduction

Inflammatory response is the process of the human body that attempts to counteract potential injurious agents, including invading viruses, bacteria, and other pathogens [1,2,3]. Inflammation can also have harmful effects on the host, which is caused by multistage biochemistry, pharmacology, and molecular control of various cells and various soluble medium, including cytokines [4,5]. Pro-inflammatory factor (NO) has been identified as a molecular that involved in the regulation process in nerves and immune system [6]. NO that is generated by activated macrophages can mediate host defense functions, such as antimicrobial and anti-tumor activities, but excess NO production induces tissue damage associated with acute and chronic inflammation. NO secreted by activated macrophages has shown antibacterial and antitumor activities, but the overproduction NO induces acute and chronic inflammation, which causes tissue damage [7]. NO is enzymatically catalyzed by nitric oxide synthases (NOSs) and formed by iNOS in macrophages. In the inflammatory response, NO plays a role by stimulating a variety of proteins of inflammatory reactions pathway, including the NF-κB and MAPKs pathways [8]. Furthermore, such specific anti-inflammatory properties are associated with certain characteristic structures, such as a main backbone of (1→6)-linked Manp and (1→6)-linked Glcp residues [9].

Naturally occurring polysaccharides are usually found in marine microorganism, marine invertebrates, such as sea cucumbers and some seaweed [10,11]. Polysaccharide structure and composition of the nature of different marine vary from species to species, but they mainly consist of fucose and sulfate with various monosaccharide and uronic acids [12,13,14,15]. *Crypthecodinium cohnii* is oleaginous heterotrophic marine dinoflagellate, in which DHA can account for more than 30% of the total fatty acids (TFA) [16,17]. It is an ideal candidate for DHA production [18]. EPA (Eicosapentaenoic acid) and DHA (Docosahexaenoic Acid) from *Crypthecodinium cohnii* have been extensively studied because of their diverse biological activities, such as antitumor, anti-inflammatory, and immunomodulatory [19]. However, the effects of immunomodulatory activity of water-soluble exopolysaccharides from *Crypthecodinium cohnii* on RAW 264.7 cell line have not been reported. The polysaccharides extracted from plants, fungi, and terrestrial microorganisms have recently attracted to researchers and consumers, due to their bioactivities and relatively low toxicity. Therefore, the detection and evaluation of polysaccharides with antitumor, anti-inflammatory, and immunostimulating bioactivities has become one of the important researches in the field of chemistry and biology [20]. This study was designed to assess the in vitro anti-inflammatory effect of exopolysaccharides extracted from *Crypthecodinium cohnii* on lipopolysaccharide-stimulated RAW 264.7 cell line.

## 2. Materials and Methods

### 2.1. Materials and Reagents

*Crypthecodinium cohnii* was supply by Third Insititude Oceanography, State Oceanic Administration. l-rhamnose, d-glucuronic, d-arabinose, dxylose, d-fructose, d-galactose, and d-mannose were purchased from Solarbio Co. (Beijing, China), while HiTrap Capto Q (16/25) and Superose 6B (10/300) were purchased from GE Co. (Fairfield, CT, USA). T-series dextrans (T-10, T-40, T-70, T-500, T-2000) as standard samples with different molecular weights were purchased from Solarbio Co. (Beijing, China). Methyl iodide (CH_3_I) was purchased from Sigma Co., Ltd. (Shanghai, China). Trifluoroacetic acid (TFA) and sodium periodate (NaIO_4_) were purchased from BLOT Co., Ltd. (Tianjin, China). 3-(4,5-Dimethylthiazol-2-y1)-2,5-diphenyltetrazolium bromide, dimethylsulphoxide (DMSO) was purchased from Sigma Co., Ltd. (Shanghai, China). RPMI-1640 medium and fetal bovine serum (FBS) was purchased from Gibco Invitrogen Co. (New York, NY, USA). Primary antibodies were purchased from CST Co. (Cell Signaling Technology, Danvers, MA, USA), secondary antibodies (HRP-IgG) were purchased from Beyotime Co. (Zhengzhou, China). NC (nitrocellulose) membrane for transfering was purchased from PALL Co. (New York, NY, USA). Other chemicals and reagents were of analytical grade.

### 2.2. Preparation, Extraction and Purification of Polysaccrides

The fermentation procedures of *Crypthecodinium cohnii* (CP) were based on previous work with some modifications [21]. Crude exopolysaccharide isolated from *Crypthecodinium cohnii* (EPCP) fermentation broth, according to a previous method in reported work [22]. Briefly, fermentation broth of *Crypthecodinium cohnii* was centrifuged (10,000× *g*, 20 min), and then the supernatant was filtered with 0.22 µm Millipore membrane. After being filtered, the deproteinization of supernatant according to Sevage method [23]. The resulting aqueous fractions without organic solvent were extensively dialyzed by distilled water for two days. The polysaccharides were precipitated by the addition of ethanol to a final concentration of 75% (*v*/*v*), after centrifugation (4000× *g*, 10 min) the precipitates were collected, the precipitates were solubilized in deionized water, and were lyophilized for 48 h. The yield of crude polysaccharides was 58.06%.

The crude polysaccharides were (10 mg) dissolved in 1 mL distilled water, centrifuged (10,000× *g*, 10 min), and then the supernatant was loaded with a column of Superose 6B (10/300) chromatography and the fraction (EPCP1) was collected. Then, the fraction (EPCP1) was purified by a column HiTrap Capto Q (16/25) [15]. After loading with EPCP1 1 mL, the column was eluted with distilled water, followed by stepwise elution with increased concentration of NaCl (0.1, 0.5, 1.0, and 1.5 M), respectively, at 1 mL/min/tube. The polysaccharide fractions were collected, dialyzed by distilled water for two days, and finally lyophilized to get the polysaccharide fraction named EPCP1-1, EPCP1-2, EPCP1-3, and EPCP1-4. The resulting elute was collected automatically and the soluble sugar content of polysaccharides were detected at 490 nm by the phenol–sulfuric acid method [24]. Finally, the fractions EPCP1-2 were lyophilized for further investigation.

### 2.3. Characterizations Analysis of EPCP1-2

The carbohydrate content was measured according to phenol-sulfuric acid colorimetric method, using glucose as a standard [24]. Protein content was measured by Bradford’s method, using bovine serum albumin (BSA) as the standard [25]. Sulfate group and the uronic acid content were determined according to the reported methods [26,27].

Gas chromatography (GC) is an efficient method to determine polysaccharides. Monosaccharide composition was analyzed based on previous researches [9,28]. For monosaccharide analysis, 5 mg EPCP1-2 was hydrolyzed with 5 mL TFA (2 M) in polytetrafluoroethylene reactor with protection of nitrogen at 110 °C for 3 h. Mole ratio calculation method was as the formula follows:

(1) Relative molar number=CB/AD,

(*A* = Peak area of standard, *B* = quality of standard, *C* = Peak area of sample, *D* = Mw (molecular weight)).

The molecular weight of EPCP1-2 was determined by using a SHIMADZU High Performance Liquid Chromatography (HPLC) analysis system (Tokyo, Japan) with a Shodex SB-804 column (*L* × *D*: 8.0 mm × 300 mm, Showa Denko K.K, Tokyo, Japan), according to our previous method [9]. Briefly, NaNO_3_ solution (0.1 mol/L) was used as the eluent at 0.8 mL·min^−1^ for 30 °C. The molecular weight was calibrated with T-series dextran standard (T-10, T-40, T-70, T-500, T-2000). The injection concentration was 2 mg/mL and volume was 15 µL.

To determine glycosyl linkages, 10–15 mg EPCP1-2 was methylated for seven times according to Needs’ method [29]. Briefly, EPCP1-2 was methylated with CH_3_I (1 mL) in distilled NaOH/DMSO. After reaction, the mixture was extracted with CCl_4_, and the organic phase was washed by distilled water. Complete methylation was confirmed by FTIR spectrum analysis, in which the disappearance or significantly decreased of the –OH band (3200–3700 cm^−1^). The per-methylated EPCP1-2 was hydrolyzed, reduced, and acetylated, as described above.

### 2.4. Cell Culture and Cell Viability Assay

Macrophage cell line, RAW 264.7 cells were maintained and cultured in RPMI 1640 (Gibco Invitrogen Co., Grand Island, NY, USA), supplemented with 10% fetal bovine serum (FBS) at 37 °C under 5% CO_2_ atmosphere. The cells were seeded into 96-well plates with the density of 5 × 10^4^ cells/well, cultured for 12 h. After being treated with EPCP (100, 200, 400, 800 μg/mL) in the presence or absence of LPS (1 μg/mL) for 36 h, MTT solution (5 mg/mL) was added to each well and incubated for another 4 h. After incubation, culture medium was removed and DMSO was added to dissolve purple precipitates. Then, plates were read at 570 nm using a Microplate spectrophotometer (Thermo Fisher, Co., Waltham, MA, USA).

### 2.5. NO Assay

Cells were plated at 5 × 10^4^ cell/well in 96-well plates, then it were incubated with or without LPS (1 μg/mL) in the presence or absence of various concentrations EPCP1-2 (50, 100, 200, 400, 800 μg/mL) for 36 h. In brief, 50 μL of cell culture medium was mixed with 50 μL of Griess reagent, the mixture was incubated at room temperature for 15 min, the absorbance at 540 nm was measured in a microplate reader. Fresh culture media were used as blanks for all of the experiments. Nitrite levels in samples were calculated with a standard sodium nitrite curve.

### 2.6. ELISA Assay of Cytokines

Cells were plated at 5 × 10^4^ cell/well in 96-well plates and then incubated with or without LPS (1 μg/mL) in the presence various concentrations EPCP1-2 (0, 50, 100, 200, 400, 800 μg/mL) of sample for 36 h. In brief, culture medium was collected for detection according to the specification in ELISA kit (Jiancheng, Nanjing, China), the absorbance was measured in a microplate reader. Fresh culture media was used as blanks for all of the experiments. *Cytokines* levels in samples were calculated with standard curves (Supplement Data S2).

### 2.7. Protein Extraction and Western Blotting

Cells were plated at 5 × 10^4^ cells/well in T-25 culture flask and then incubated with or without LPS (1 μg/mL) in the presence of various concentrations EPCP1-2 (0, 100, 200, 400, 800 μg/mL). After incubation, the cells were collected by centrifugation and washed twice with cold PBS. The cells were lysed in RIPA buffer with 1 mM PMSF (phenylmethylsulfonyl fluoride) on ice for 15 min. The cell lysates were determined as the previous research reported [29]. Finally, the immune-active proteins were separated by Western blot.

### 2.8. Statistical Analysis

All data were expressed as means ± S.D. *n* = 6. Significant differences among the groups were determined using the unpaired *t*-test. A value of * *p* < 0.05 and ** *p* < 0.01 were considered to be statistically significant.

## 3. Result

The EPCP were purified by Superose 6B (10/300) chromatography and HiTrap Capto Q (16/25). Four major fractions were isolated with 0.1 mol/L, 0.5 mol/L, 1.0 mol/L, 1.5 mol/L NaCl. All of these fractions possess anti-inflammatory properties in a range of tests, but one of these fractions possesses the most potent.

### 3.1. Purification of Hydrolysates of Exopolysaccharide of Crypthecodinium Cohnii (EPCP)

**Figure 1:**
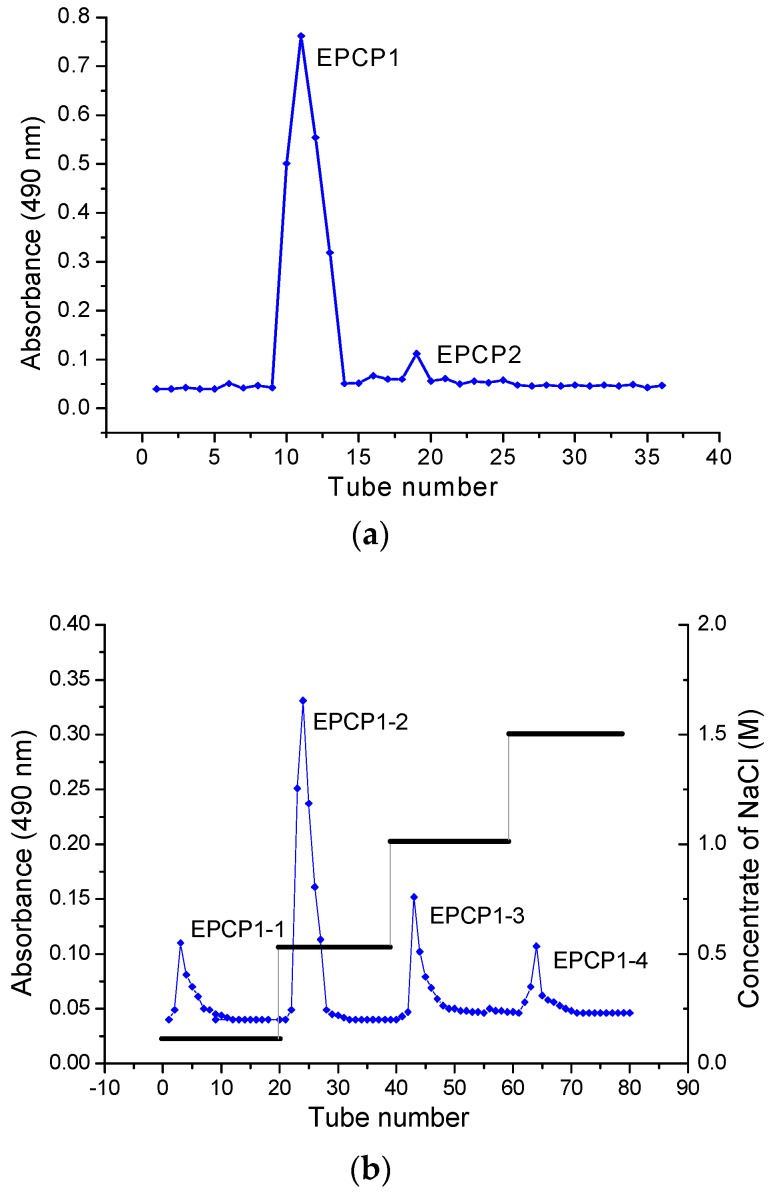
(**a**) Stepwise elution curve of crude *Crypthecodinium cohnii* (EPCP) on size-exclusion chromatography column of Superose 6B (10/300) and (**b**) elution curve of polysaccharide fractions from Superose 6B on anion-exchange chromatography column of HiTrap Capto Q (16/25).

**Figure 2:**
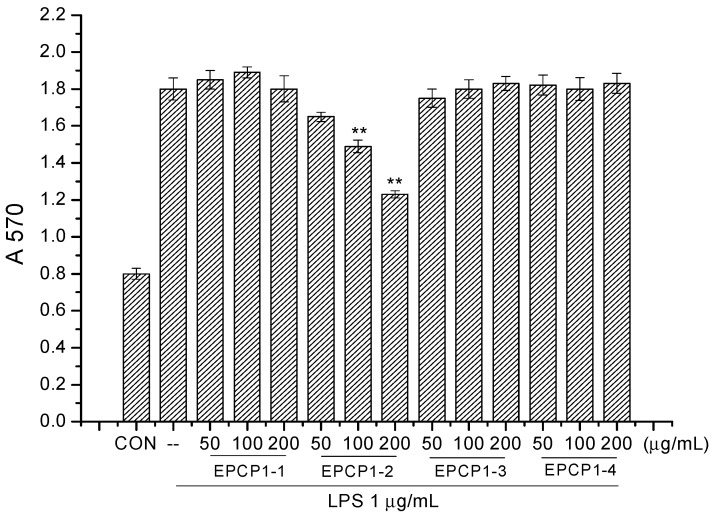
Anti-inflammatory effect of EPCP1 (EPCP1-1, EPCP1-2, EPCP1-3, EPCP1-4) on RAW 264.7 induced by LPS. (Results are expressed as the mean ± S.D. of three separate experiments). Statistical significance was tested using a Student’s *t*-test. EPCP group ** *p* < 0.01.

**Figure 3:**
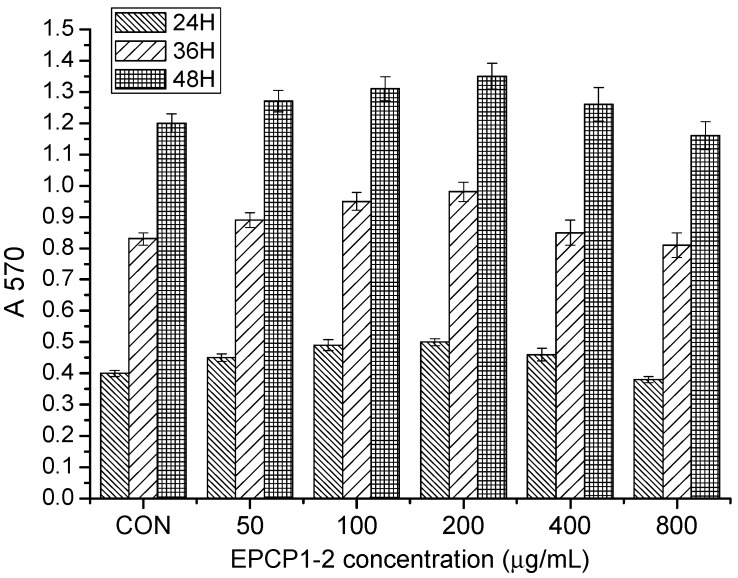
Cytotoxicity assay of EPCP1-2 (50, 100, 200, 400, 800 μg/mL) on RAW 264.7. (Results are expressed as the mean ± S.D. of three separate experiments). Statistical significance was tested using a Student’s *t*-test.

EPCP was firstly separated through a gel chromatography of Superose 6B (10/300) column and anion-exchange column of HiTrap Capto Q (16/25). Two fractions were clearly separated, coded as EPCP1, EPCP2. The proportion of EPCP1 was 98%. EPCP1 was eluted consecutively by 0.1, 0.5, 1.0 and 1.5 mol/L NaCl, coded as EPCP1-1, EPCP1-2, EPCP1-3 and EPCP1-4, respectively. As shown in the profile of gel filtration chromatograms (Figure 1a), two fractions were clearly separated. The anion-exchange chromatogram of the four fractions is shown in (Figure 1b). As Figure 2 showed, only EPCP1-2 had strong activity of anti-inflammatory. In order to explore the cytotoxicity of EPCP1-2 on RAW 264.7 cell line, MTT assay was conducted to test the cell proliferation. As shown by Figure 3, EPCP1-2 promoted proliferation of RAW 264.7 at low concentrations (50, 100, 200 μg/mL). With increase of EPCP1-2 concentration the proliferation showed decrease gradually. But generally, when compared with the control group (0 μg/mL EPCP1-2), the treatment group with EPCP1-2 (400, 800 μg/mL) showed no significant decrease. The results indicated EPCP1-2 exhibited anti-inflammatory effect without cytotoxicity on RAW 264.7 cell line.

### 3.2. Basic Properties of EPCP1-2

**Figure 4:**
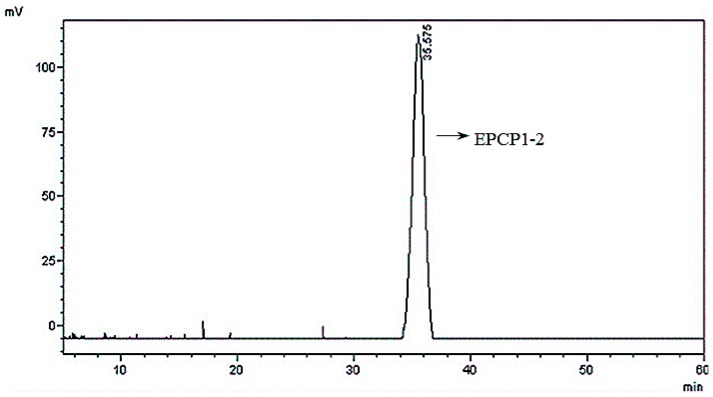
High Performance Liquid Chromatography (HPLC) analysis of EPCP1-2.

The ratio of the peak area was 99.5% (Figure 4). The extraction process had a relatively high reproducibility, thus the EPCP1-2 molecular weight was estimated to be 82.5 kDa (Mw = 82.52, Mn = 74.86), which was calibrated by the dextran calibration curve (T-series dextran). (Mw: weight-average molecular weight, Mn: number average molecular weight).

No absorption at 280 nm and 260 nm was detected indicated the EPCP1-2 did not contain proteins or nucleic acids. EPCP1-2 was free of proteins, which was detected by Lowry assay. No uronic acid was detected using the m-hydroxydiphenol method with d-glucuronic acid as the standard. The phenol sulfuric acid assay suggests that total sugar content of EPCP1-2 is 92%. These results demonstrate that EPCP1-2 is a neutral polysaccharide.

From monosaccharide and absolute configuration analyses, EPCP1-2 was hydrolyzed, reduced, and acetylated. GC spectrum indicated that EPCP1-2 was composed of rhamnose, mannose, glucose, and galactose with a molar ratio of 1, 17.13, 3.45 and 7.43 (Supplement Data S1).

**Figure 5:**
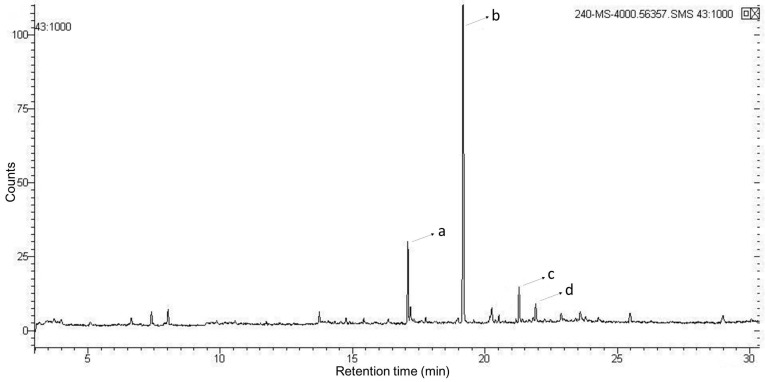
Total ion gas chromatogram of methylation patterns of EPCP1-2 using GC-MS. Note: signals a–d represent 2,4-Me2-Gala*p*, 2,3,4-Me3-Man*p*, 2,3,4-Me3-Glc*p*, respectively. The *x*-axis represents retention time in minutes; the *y*-axis represents signal intensity by counts.

Methylation analysis by GC-MS was used to investigate the glycosyl linkages of EPCP1-2. Figure 5 and Table 1 showed the three types of linkages, were, namely, 2,4-Me2-Gala*p*, 2,3,4-Me3-Man*p*, 2,3,4-Me3-Glc*p*, 2,3,4,6-Me3-Rha, at a percentage of 23.21%, 60.56%, 11.11%, 3.02%. The molar ratio of monosaccharide agrees with the percentages of methylated sugar residues in EPCP1-2. These findings indicated that the repeating unit of EPCP1-2 consists of 1,6-linked-Mannose, 1,3,6-linked-Galactose, 1,6-linked-glucose, and *t*-l-Rha*p*.

### 3.3. Effect of EPCP1-2 on LPS Induced NO Production from RAW 264.7

**Figure 6:**
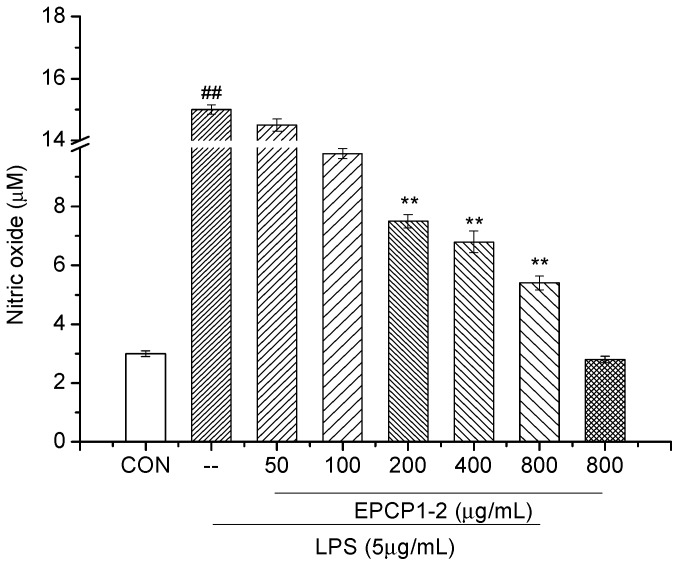
Effects of EPCP1-2 on pro-inflammatory factor (NO) production in RAW 264.7. (Results are expressed as the mean ± S.D. of three separate experiments). Statistical significance was tested using a Student’s *t*-test. EPCP1-2 group ** *p* < 0.01; LPS group (positive control) ^##^ *p* < 0.01.

In order to identify the anti-inflammatory effects of EPCP1-2, we investigated the inhibitory effects of EPCP1-2 on NO production. As Figure 6 showed, when RAW 264.7 cells were stimulated with LPS, nitrite was produced as a biomarker of NO. Various concentration of EPCP1-2 (50, 100, 200, 400, 800 μg/mL) were tested on RAW 264.7 cells with or without stimulation of LPS. Treatment with EPCP1-2 and without LPS stimulation had a mild inhibitory effect on NO production, the inhibition only happened at the highest concentration (800 μg/mL). EPCP1-2 reduced NO production in a concentration-dependent manner after treated for 36 h (Figure 6), with a maximum effects of 64% NO reduction with 800 μg/mL of EPCP1-2, and exhibited the strong inhibitory properties and the lack of any cytotoxic effects. In addition, there was no significant difference between the negative control group and the control group, so the treatment of 800 μg/mL dose EPCP1-2 did not inhibited NO reduction of the cells.

### 3.4. Effect of EPCP1-2 on LPS Induced Cytokines Production from RAW 264.7

IL-1β is produced by activated monocytes and macrophages, which can promote the proliferation and secretion of B lymphocytes in the immune response. Tumor necrosis factor (TNF-α) is produced by monocytes, macrophages, and T lymphocytes, playing an important role in the immune defense and immune stability of the body. The physiological function of IFN-γ is macrophage activation, immune response, and host defensive immunity, is an important factor that mediates cellular immunity and humoral immunity. In order to investigate the effect of EPCP1-2 on the secretion of inflammatory cytokines in RAW 264.7, the level of cytokines in the cell culture medium was measured by ELISA assay. As Table 2 showed, a certain concentration (200, 400, 800 μg/mL) of EPCP1-2 could inhibit the secretion of inflammatory cytokines and showed a certain dose-dependent. In addition, there was no significant difference between the negative control group and the control group, so that the treatment of 800 μg/mL dose EPCP1-2 did not cause excitation or inhibition to the cells.

### 3.5. Effect of EPCP1-2 on Expression of Phosphorylation and Total TLR4

**Figure 7:**
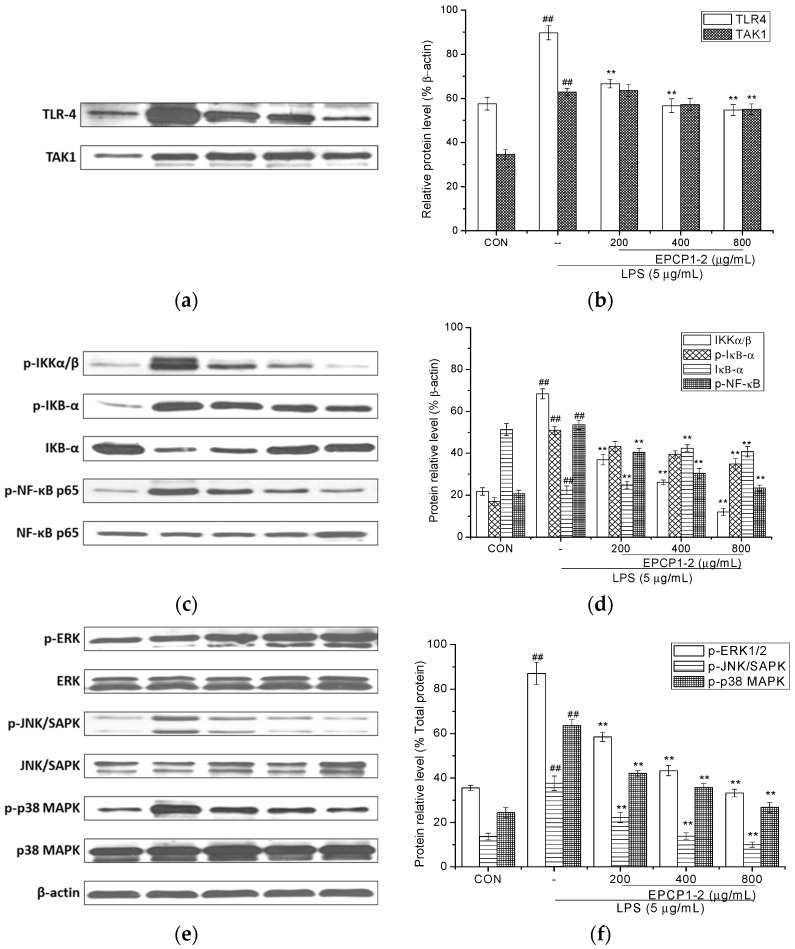
Inhibitory effect of EPCP1-2 isolated from *Crypthecodinium cohnii* on the NF-κB protein activation in LPS-induced RAW 264.7 cells. Cells were stimulated by LPS (5 μg/mL) for 1 h with or without the presence of EPCP1-2 (200, 400, 800 μg/mL). The protein levels of IκB-α and NF-κB were determined using ECL (electrochemiluminescence) immunoblotting method. (**a**) TLR4 protein and TAK1 protein expression were examined by Western blotting analysis. (**b**) Densitometric analysis showed the effects of EPCP1-2 on LPS-induced expressions of TLR4 and TAK1 proteins. (**c**) NF-κB p65 signal relative proteins expression was examined by Western blotting analysis. (**d**) Densitometric analysis showed the effects of EPCP1-2 on LPS-induced expressions of NF-κB p65 signal relative proteins. (**e**) Densitometric analysis showed the effects of EPCP1-2 on LPS-induced expressions of IKKα/β, IκB-α and NF-κB proteins. (**f**) Densitometric analysis showed the effects of EPCP1-2 on LPS-induced expressions of ERK1/2, JNK/SAPK, p38 MAPK proteins. (Results are expressed as the mean ± S.D. of three separate experiments). Statistical significance was tested using a Student’s *t*-test. EPCP1-2 group ** *p* < 0.01; LPS group (positive control) ^##^ *p* < 0.01.

TLRs are recognized as a wide variety of pathogen-associated molecular patterns (PAMP) in viruses, bacteria, fungi, and cell, they play an important role in immune-regulatory functions [30]. Previous research reported that TLRs are related to NF-κB, JNK, and p38 MAPK. Their downstream signaling pathways are related to induction and secretion of cytokines, chemokines, and other inflammatory mediators [31]. RAW 264.7 culture were treated with different concentrations of EPCP1-2 (200, 400, 800 μg/mL) and stimulated with 5 μg/mL of LPS for 6 h. Western blotting showed that 5 μg/mL LPS induced strong activation of TLR4, and treated with EPCP1-2 (200, 400, 800 μg/mL) showed a reduced of TLR4 expression with dose dependent. Moreover, we investigated the effect of EPCP1-2 (200, 400, 800 μg/mL) on the interaction of TLR4 with adapter molecule TAK1 in LPS-induced RAW 264.7. As shown in Figure 7A, LPS stimulation of RAW 264.7 caused an increase in TAK1 expression, which was significantly inhibited by EPCP1-2. In a conclusion, the formation of TLR4/TAK1 complex significantly increased after LPS stimulation. Cells that were treated with different concentrations EPCP1-2 showed a reduction in the expression of the TAK1 protein.

### 3.6. Effect of EPCP1-2 on Expression of Phosphorylation and Total NF-κB

To further examine the effects of EPCP1-2 on the up-stream regulators of immune signaling, the effects were determined on IκB-α phosphorylation by Western blot. As shown in Figure 7c, LPS induced the phosphorylation of IκB-α, which was inhibited by the treatment of EPCP1-2. These results were consistent with the anti-inflammatory effect of EPCP1-2 that is mediated by the inhibition of IκB-α phosphorylation. Therefore, there was dose-dependent effect found. NF-κB is an important transcription factor complex that controls the expression of pro-inflammatory mediators, such as iNOS and NO [32]. To investigate the molecular mechanism underlying the mediated inhibition of iNOS and NO expression, western blot analysis was performed to quantify the presence of NF-κB in its phosphorylated states as an indirect measure for NF-κB transcriptional activity. As shown in Figure 7c, treatment with LPS increased the phosphorylation of NF-κB p65, and EPCP1-2 inhibited these LPS-induced phosphorylations.

### 3.7. Effect of EPCP1-2 on Expression of MAPKS Phosphoorylation and JNK/SAPK Phosphoorylation Induced by LPS

LPS induction of cytokine expression occurs via activation of MAPK and ERK phosphorylation following binding to TLR4 [33]. The MAPK signaling pathways have been shown to modulate the synthesis and release of pro-inflammatory mediators and cytokines in macrophages that are stimulated by LPS [34]. To understand whether the anti-inflammatory activities of EPCP1-2 are mediated through the MAPK pathway, the effects of EPCP1-2 on LPS-induced phosphorylations of p38 MAPK and ERK1/2 were examined by Western blot. The effect of EPCP1-2 on the activation of MAPK pathways was examined by quantitating MAPK in the phosphorylated form using specific antibodies. RAW 264.7 cells were treated with different concentrations of EPCP1-2 and stimulated with 1 μg/mL of LPS for 6 h. As shown in Figure 7e, the activation of MAPK (p38 MAPKand ERK1/2) and JNK signaling did occur in RAW 264.7 cells compared with the control group. LPS induced p38 ERK and JNK phosphorylation was attenuated by EPCP1-2. Co-treatment with increasing concentrations of EPCP1-2 suppressed LPS-induced phosphorylation of p38, ERK 1/2, and JNK in dose-dependent manner. The amounts of non-phosphorylated p38 ERK 1/2 and JNK were treated with or without LPS and EPCP1-2. As the results shown in Figure 7e, the anti-inflammatory activity of EPCP1-2 was mediated by inhibition of the phosphorylation of p38, ERK1/2, JNK induced by LPS.

## 4. Discussion

Macrophages play a key role in inflammation and immune responses by secreting chemokines and cytokines, recognizing and phagocytizing pathogens, processing antigens, and repairing tissue damage [35]. LPS can activate macrophages and initiate inflammatory and immune responses, which induces the production of inflammatory cytokines, including TNF-α and IL-8 [36,37]. During inflammation, LPS induces the production of pro-inflammatory mediators, such as NO and PGE2 by modulating iNOS and COX-2 expression, respectively [38]. NO is a key inflammatory mediator, and excessive NO production occurs in both chronic and acute inflammation [39]. Furthermore, NO and iNOS production levels significantly correlate with the degree of inflammation [40]. To further investigate the mechanisms that are underlying EPCP1-2 activity, the expression levels of TLR4, TAK1, and NF-κB were examined by Western Blot. Our present studies showed that EPCP1-2 exerted potent inhibition effects of NF-κB pathway via mediating TLR4-TAK1 complex signaling pathway.

**Figure 8:**
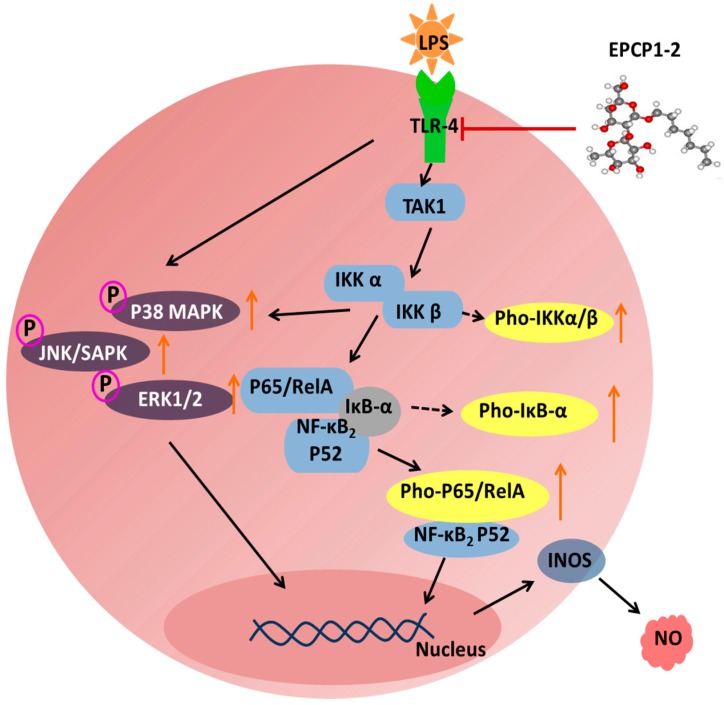
Schematic diagram of the propose target for the anti-inflammatory effects of EPCP1-2 in the RAW 264.7 cells stimulated by LPS, potentially leading to the inhibition of the pro-inflammatory cytokines and related mediators.

Toll like receptors (TLRs) have been generally considered as therapeutic targets of autoimmune diseases [41]. TLR4 is involved in inflammation and injury of renal. As previous research showed, TLR4-deficient lupus prone mice demonstrated a more global decrease in immune responses, cytokine production, autoantibody production, and attenuation in renal damage [42,43]. In this research, we identified the potent pro-inflammatory effects on LPS-induced RAW 264.7, confirming the correlation to the inflammation of LPS induced in previous findings RAW 264.7. According to our research, the protein expression of TLR4 was upregulated with LPS induction, treatment with EPCP1-2 showed inhibiting effect on expression of TLR4 in LPS-induced RAW 264.7. Moreover, treatment with EPCP1-2 showed inhibiting effect on TLR4 expression with a dose-dependent manner. EPCP1-2 may competitive suppress the binding of LPS and receptor protein TLR4, which might be a mechanism of anti-inflammatory activity of EPCP1-2 on LPS-induced RAW 264.7. In this research, TLR4 has been identified as the cellular receptor that transduces LPS signaling. The binding of LPS and receptor protein TLR4 triggers intracellular signaling, including the NF-κB pathway. NF-κB was involved in initiating the transcription of genes that are involved in mediating the fibrotic and inflammatory process in RAW 264.7 [44,45]. The results in our study indicated that EPCP1-2 inhibited LPS-activated NF-κB pathway through downregulating IκB expression and NF-κB-P65 nuclear translocation, as shown in Figure 8. These results (Figure 7c,d) showed that EPCP1-2 suppresses LPS-stimulated NF-κB activation by preventing the degradation of IκB-α.

The results in our research suggested that EPCP1-2 contains a long main backbone of (1→6)-linked Man*p* and (1→6)-linked Gal*p* residues and (1,3→6)-linked Man*p* as the branch, which inhibited the activation of RAW 264.7 through the IκB-NF-κB signal transduction pathway, thus elucidating the relationship between its chain conformation and anti-inflammation activity. The EPCP1-2 fractions with molecular weights about 82.5 kDa showed significant immunomodulatory effects, which agreed with the previous research, in which the water-soluble polysaccharide fraction showed immunomodulatory effects with molecular weight was ≈100 kDa [46]. The bioactivity of EPCP1-2 may depend on the composition of main backbone (1→6)-linked Man*p*, which serves as the main backbone of a neutral heteropolysaccharide extracted from *Auricularia polytricha* [47].

## 5. Conclusions

EPCP1-2 was an extracellular polysaccharide extracted from *Crypthecodinium cohnii* which contained a long main backbone of (1→6)-linked Man*p* and (1→6)-linked Gal*p* residues and (1,3→6)-linked Man*p* as the branch. EPCP1-2 had a high capacity to inhibit macrophage proliferation, downregulation of the expression of TRL4, TAK1, MAPKs, and NF-κB protein, and acted as an anti-inflammatory agent through macrophage suppression. In conclusion, our findings demonstrated that the EPCP1-2 extracted from *Crypthecodinium cohnii* against inflammation was considered to be a potent regulatory element in TLR4-TAK1 complex mediated MAPK and NF-κB signaling pathways, as Figure 8 showed.

## Figures and Tables

**Table 1:** Gas chromatography (GC)/MS data on methylated sugar residues in EPCP1-2.

Peak No.	Methylated Sugar Residue	Retention Time (min)	Linkage Type
a	2,4-Me2-Gala	17.085	1,3-6-d-Galac*p*
b	2,3,4-Me3-Man	19.175	1,6-d-Man*p*
c	2,3,4-Me3-Glc	21.294	1,6-d-Glc*p*
d	2,3,4,6-Me3-Rha	21.911	*t*-l-Rha*p*

**Table 2:** Effect of EPCP1-2 on the protein expression of cytokines (IL-1β, IFN-γ, TNF-α) in RAW 264.7 cells. (Results are expressed as the mean ± S.D. of three separate experiments). Statistical significance was tested using a Student’s *t*-test. EPCP1-2 group ** *p* < 0.01, LPS group (positive control) ^##^ *p* < 0.01.

Group	EPCP1-2 (μg/mL)	LPS (μg/mL)	IL-1β (ng/L)	IFN-γ (ng/L)	TNF-α (ng/L)
Control	-	-	175.5 ± 5.8	200.3 ± 5.1	187.8 ± 3.5
Positive control	-	5	365.3 ± 9.1 ^##^	445.6 ± 3.5 ^##^	315.3 ± 6.4 ^##^
Treatment group	200	5	320.3 ± 5.3 **	395.4 ± 5.6 **	300.5 ± 9.2 **
	400	5	284.7 ± 6.2 **	318.6 ± 8.1 **	281.3 ± 7.4 **
	800	5	214.1 ± 4.4	245.4 ± 6.7	226.4 ± 7.3
Negative control	800	-	180.5 ± 7.5	204.9 ± 4.3	195.2 ± 6.3

## Supplementary Materials

The following are available online at www.mdpi.com/1660-3397/15/12/376/s1, Supplement Data S1: GC spectrum of monosaccharide composition of EPCP1-2; Supplement Data S2: Standard curve of cytokines protein expression by Elisa (**a**) IL-1β (**b**) IFN-γ (**c**) TNF-α.
