# Supplementary material for: The Anti-Inflammatory Effect and Structure of EPCP1-2 from Crypthecodinium cohnii via Modulation of TLR4-NF-κB Pathways in LPS-Induced RAW 264.7 Cells
Source: Mar Drugs. 2017 Dec 1;15(12):376. doi: 10.3390/md15120376 (PMC5742836; doi:10.3390/md15120376)
Supplement: Supplementary file 1 [file marinedrugs-15-00376-s001.pdf]

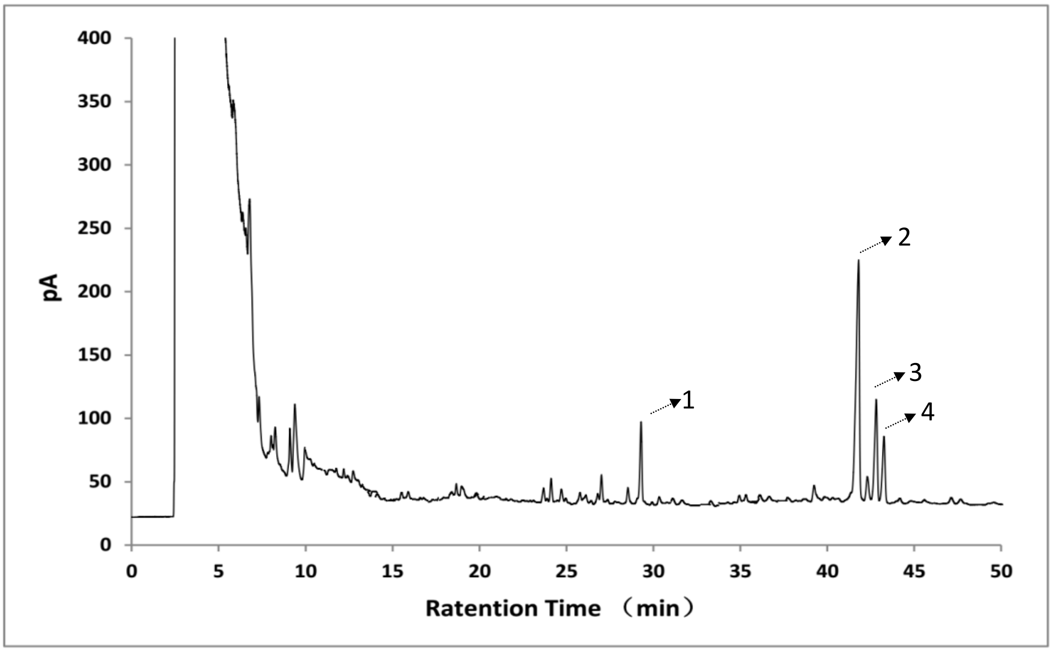

Supplement data 1. GC spectrum of monosaccharide composition of EPCP1-2.
Note:1: rhamnose; 2: mannose; 3: galactose; 4: glucose.

(a)

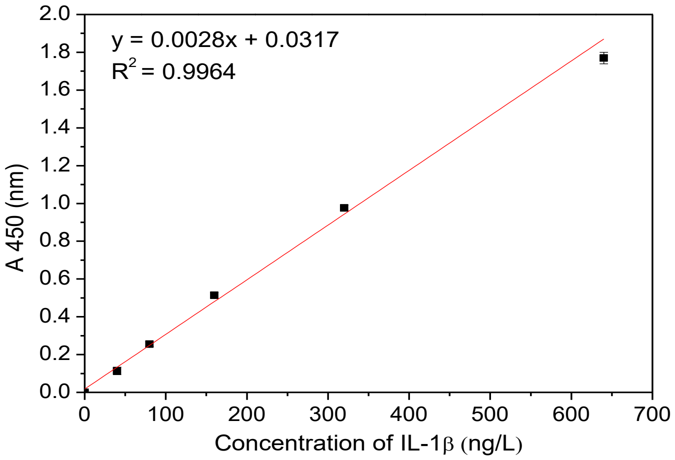

(b)

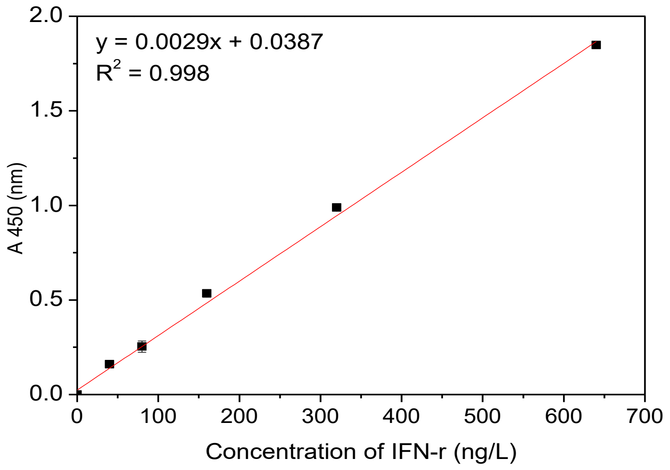

(c)

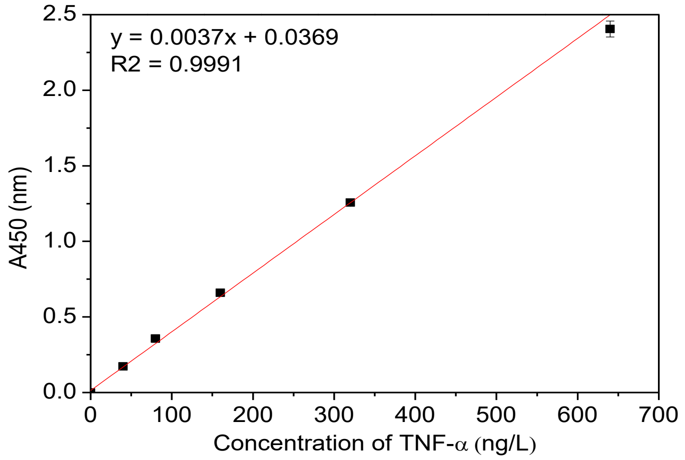

Supplement data 2. Standard curve of cytokines protein expression by Elisa (a) IL-1 β (b) IFN- γ (c) TNF- α
